# Emerging Roles of Extracellular Vesicles in Alzheimer’s Disease: Focus on Synaptic Dysfunction and Vesicle–Neuron Interaction

**DOI:** 10.3390/cells12010063

**Published:** 2022-12-23

**Authors:** Martina Gabrielli, Francesca Tozzi, Claudia Verderio, Nicola Origlia

**Affiliations:** 1 CNR-Institute of Neuroscience, 20854 Vedano al Lambro, Italy; 2 Bio@SNS Laboratory, Scuola Normale Superiore, 56124 Pisa, Italy; 3 CNR-Institute of Neuroscience, 56124 Pisa, Italy

**Keywords:** Alzheimer’s disease, extracellular vesicles, synaptic dysfunction, extracellular vesicle–neuron interaction, beta amyloid, tau protein

## Abstract

Alzheimer’s disease (AD) is considered by many to be a synaptic failure. Synaptic function is in fact deeply affected in the very early disease phases and recognized as the main cause of AD-related cognitive impairment. While the reciprocal involvement of amyloid beta (Aβ) and tau peptides in these processes is under intense investigation, the crucial role of extracellular vesicles (EVs) released by different brain cells as vehicles for these molecules and as mediators of early synaptic alterations is gaining more and more ground in the field. In this review, we will summarize the current literature on the contribution of EVs derived from distinct brain cells to neuronal alterations and build a working model for EV-mediated propagation of synaptic dysfunction in early AD. A deeper understanding of EV–neuron interaction will provide useful targets for the development of novel therapeutic approaches aimed at hampering AD progression.

## 1. Introduction

Alzheimer’s disease is the most common cause of dementia, affecting more than 50 million people worldwide. It is a progressive degenerative encephalopathy causing deterioration in memory, thinking, behaviour, and ability to perform daily activities, and it leads to death. Neuropathological hallmarks of the disease are amyloid beta (Aβ) and tau protein aggregate formation, as well as loss of synapses and neurons [1]. These processes are accompanied by an abnormal response in microglia, the innate immunity cells resident in the brain, which are involved in disease progression [2]. Activated astrocytes, oligodendrocytes, and components of the neurovascular unit also play a role [3]. The neuropathological hallmarks of AD are evident at advanced stages of the disease, first in specific brain regions and then in progressively larger brain areas [4,5,6,7]. By contrast, extensive literature indicates that the first mechanism affected in the disease is functional alteration of the synapses [8,9], which underlies the appearance of clinical symptoms many years later.

Medicine is currently unable to cure AD or to interrupt its progression. Available drugs only treat AD symptoms, temporarily helping memory and thinking problems [10], whereas effective treatments would target the very early stages of the disease, close to synaptic dysfunction onset. For this reason, understanding how synaptic alteration starts and propagates through the brain represents a crucial issue in AD research.

AD pathology progression has been associated with the spreading of Aβ and tau proteins from neuron to neuron throughout the affected brain in animal models [11,12,13,14,15,16]. Importantly, soluble oligomeric forms of these misfolded proteins, before forming extracellular aggregates, have been identified as a cause of synapse dysfunction and neurotoxicity and involved in glia activation. In this context, recent evidence has implicated brain cell-derived extracellular vesicles (EVs) carrying Aβ and tau forms in the onset and propagation of synaptic alterations in AD [15,17,18].

EVs are membrane structures capable of shuttling all sorts of active molecules (proteins, lipids, and nucleic acids) from a donor cell to specific target cells, thus being involved in cell-to-cell communication. EVs can directly bud from the plasma membrane (microvesicles) or be generated in the endocytic compartment (exosomes). Due to technical limitations in isolating and distinguishing EVs depending on their mechanism of biogenesis, the currently recognized nomenclature identifies EVs according to their dimensions, distinguishing medium-large/large EVs (>200 nm) and small EVs (<200 nm) [19].

A large body of evidence indicates that EVs released by neurons and glial cells contain Aβ and tau proteins in transgenic AD mice as well as in culture AD models [15,17,20,21,22,23,24,25,26,27]. The presence of misfolded proteins among EV cargo has been validated in EVs extracted from both body fluids (cerebrospinal fluid (CSF) and plasma) and brain tissue of AD patients [18,20,21,23,28,29,30,31,32]. Encapsulation into EVs protects Aβ and tau from degradation, enhancing their pathogenic action and promoting their diffusion through the brain [15,17,21,23,33].

In this review, we will summarize the current knowledge regarding the role of EVs in the rise and propagation of synaptic alterations in AD, especially as carriers of Aβ and tau proteins, and analyse the possible mechanisms of interaction of EVs with neurons. Findings will be presented based on the cellular source of EVs, to enlighten possible cell-specific EV functions.

## 2. Synaptic Dysfunction in AD

Synaptic failure is a major determinant of AD [9]. In fact, synapse loss better correlates with cognitive impairment in the disease, rather than with the number of plaques and fibrillary tangles, the grade of neuronal loss, or the extent of gliosis [34]. Furthermore, the synapse is where Aβ peptides are produced and where their effects are targeted [35]. Numerous lines of evidence locate alterations in synaptic function and synapse degeneration in the earliest phases of AD, before the accumulation of misfolded protein aggregates and before neuronal loss [9,34,36]. 

Aβ oligomers appear to be pivotal players in AD pathogenesis and are particularly active on the synapse [1], being able to alter calcium homeostasis and reduce excitatory synapses’ strength and plasticity. Aβ oligomers impair long-term potentiation (LTP) and long-term depression (LTD), long-term forms of synaptic plasticity thought to underlie learning and memory, in a concentration-dependent manner, leading to cognitive deficits [37]. In particular, high levels of Aβ oligomers affect excitatory synaptic transmission by decreasing the number of surface AMPA and NMDA receptors, dismantling dendritic spines. Compensatory inhibitory responses in circuits involved in learning and memory are also part of AD early synaptic alterations [38]. Changes in synaptic function may cause network instability and lead to synchronous (epileptiform) activity [37]. These effects are not restricted to animal models, as AD patients display aberrant increases in neuronal activity during memory encoding in hypometabolic regions, and early-onset familiar cases show epileptic brain activity.

Pathogenic tau can also be detrimental for the synapse in different ways: it reduces the mobility and release of synaptic vesicles, decreases the number of glutamatergic receptors, affects dendritic spine maturation, disrupts mitochondrial transport and function in the synapses, and promotes the phagocytosis of synapses by microglia [39]. Similarly to Aβ species, tau oligomeric forms are more toxic to the synapse than tau fibrils, impairing LTP and dendritic spine density and maturation, and causing memory deficits [40,41,42]. The regulation of tau by specific phosphorylations recently emerged as crucial for its role at the synapse [43].

Interestingly, several studies support the idea that small oligomeric forms of Aβ and tau may act synergistically in causing synaptic deficits [44,45,46,47,48,49]. 

Finally, abnormal complement-mediated synaptic pruning seems to play a role in early synaptic loss in AD: complement factors have been found to be necessary for the expression of Aβ effects on the synapse and their depletion to rescue cognitive deficits in APP/PS1 AD mice [50,51,52,53]. Accordingly, it has been proposed that Aβ oligomers enhance the expression of C3 in microglia and astrocytes, driving synapse tagging for elimination [53].

## 3. EVs and Synaptic Dysfunction in AD

**Figure 1:**
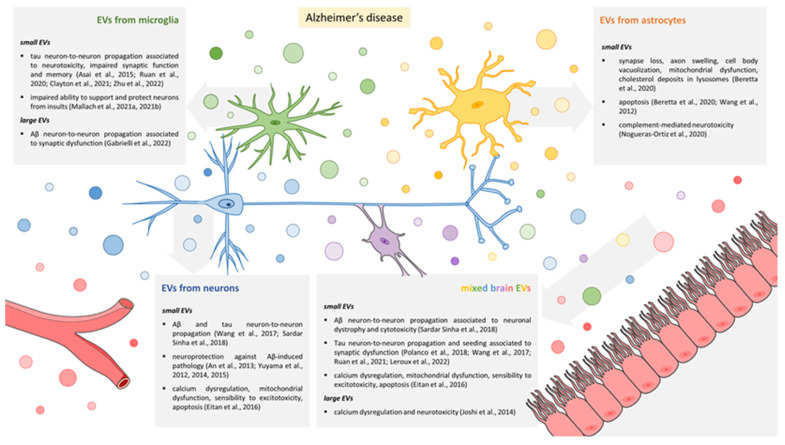
Effects of EVs released by brain cells on neuronal function in AD. Graphic summary of the effects of EVs from different brain cell types on neuronal function in AD pathology. Parts of the figures were drawn by using pictures from Servier Medical Art, licensed under a Creative Commons Attribution 3.0 Unported License (https://creativecommons.org/licenses/by/3.0/; access date 20 November 2022). Asai et al., 2015 [15], Ruan et al., 2020 [54], Clayton et al, 2021 [55], Zhu et al., 2022 [56], Mallach et al., 2021a, 2021b [57,58], Gabrielli et al., 2022 [17], Wang et al., 2017 [59], Sardar-Sinha et al., 2018 [26], An et al., 2013 [60], Yuyama et al., 2012, 2014, 2015 [61,62,63], Eitan et al., 2016 [25], Polanco et al., 2018 [64], Ruan et al., 2021 [18], Leroux et al., 2022 [65], Joshi et al., 2014 [21], Beretta et al., 2020 [66], Wang et al., 2012 [67], Nogueras-Ortiz et al., 2020 [68].

In the brain, EVs are secreted by all cell types, including glial cells and neurons. They can affect the synapse and propagate synaptic alterations among connected cells in a way that poses them as attractive therapeutic targets. The effects of EVs released by different cell types on neuronal function in the context of AD are summarized in Figure 1. 

### 3.1. EVs Released by Microglia

Microglia, the innate immunity cells resident in the brain, are essential regulators of synaptic function and neuronal network formation [2]. They react to the smallest stimulus, being able to assume a various and complex range of activation states [69]. When brain homeostasis is endangered, microglia orchestrate a weighted response to re-establish the status quo [70]. Under sustained brain alterations, as in the case of AD, microglia undergo a neurodegenerative/disease-associated (MGnd/DAM) phenotypic change [71,72,73] and become determinants of disease pathogenesis [74]. Accumulating evidence suggests that DAM might play a positive and protective role in early disease pathology while in late AD stages, DAM might become dysregulated and accelerate the disease [75,76]. The central role of microglia in AD [77] and related synaptic dysfunction [15,50,78,79] has long been known, and the fact that many AD risk genes pertain to microglia and their functions strengthens this concept [77,80,81,82,83].

Studies from our group and others indicated that EVs released by microglia can influence synapse formation, inducing new spines at sites of contact with neurons [17], emulating what happens at microglia–synapse contact sites [84,85]. In addition, cultured neurons exposed to large EVs derived from primary rat microglia show an increase in miniature excitatory postsynaptic current (mEPSC) frequency in a dose-dependent manner, without changes in their amplitude [86]. Analysis of paired-pulse recordings showed that EVs mostly act at the presynaptic site, increasing neurotransmitter release probability [86] and the availability of synaptic vesicles for release [87]. The effect of microglial EVs was confirmed in vivo in cingulate cortex slices from the mouse brain [88], as well as in the rat visual cortex, where injection of large EVs caused an acute increase in the amplitude of field potentials evoked by visual stimuli [86]. Furthermore, a subsequent study showed that microglial large EVs are enriched in endocannabinoids, which are capable of inducing a decrease in miniature inhibitory post-synaptic currents (mIPSCs) targeting CB1 receptors on GABA-ergic cells [89]. More importantly, when microglia are exposed to an inflammatory stimulus, they become detrimental for synaptic function by releasing EVs, which are enriched in a set of miRNAs that regulate the expression of key synaptic proteins. In particular, we demonstrated that large EVs from microglia activated by a cocktail of pro-inflammatory cytokines transfer miR-146a-5p to neurons, leading to the suppression of synaptotagmin 1, a pre-synaptic protein, and neuroligin 1, a postsynaptic adhesion protein that maintains synaptic stability and plays a key role in dendritic spine formation, with detrimental effects on synaptic strength and dendritic spine remodelling [90]. Similar effects on dendritic spines are mediated by small EVs released by primary mouse microglia inflamed after saturated fatty acid palmitate exposure, a model of a high-fat diet [91]. These findings link inflammatory microglia and enhanced EV production to loss of excitatory synapses. 

This link was recently confirmed in models of AD, where the implication of large and small microglial EVs in synaptic dysfunction has also been demonstrated.

In a seminal paper, Asai and colleagues used a model of rapid tau propagation from the entorhinal cortex–EC to the dentate gyrus of the hippocampus–DG, together with in vitro systems, to demonstrate that tau propagates between these two regions, causing reduced excitability in DG cells as well as cytopathic changes [15]. Interestingly, tau spreading was limited by both microglia depletion and EV synthesis inhibition [15], while the working and contextual memory deficits were rescued in the P301S tau transgenic mouse model by treatment with a P2 × 7 receptor antagonist, which blocks EV release from microglia [54]. 

In line with these findings, microglial immune receptor Trem2 deletion in mice (*Trem2* KO), a condition known to aggravate tau pathology, enhances tau spreading from the EC to the hippocampus through small EVs, which coincides with impaired synaptic function and memory behaviour [56]. *TREM2* is in fact a risk gene for AD and an important regulator of microglia response to pathological changes. *R47H* heterozygous mutation of *TREM2* is linked to late onset AD, and small EVs released by microglia-like cells differentiated from iPSCs in patients carrying this variant (*R47Hhet* EVs) have been characterized. These EVs contain more inflammatory and DAM-associated proteins than common variant EVs (Cv EVs) [57]; they lose the ability to promote neurite outgrowth and neuronal metabolism; and lose their protective functions against AD-related insults to neurons [58]. 

**Figure 2:**
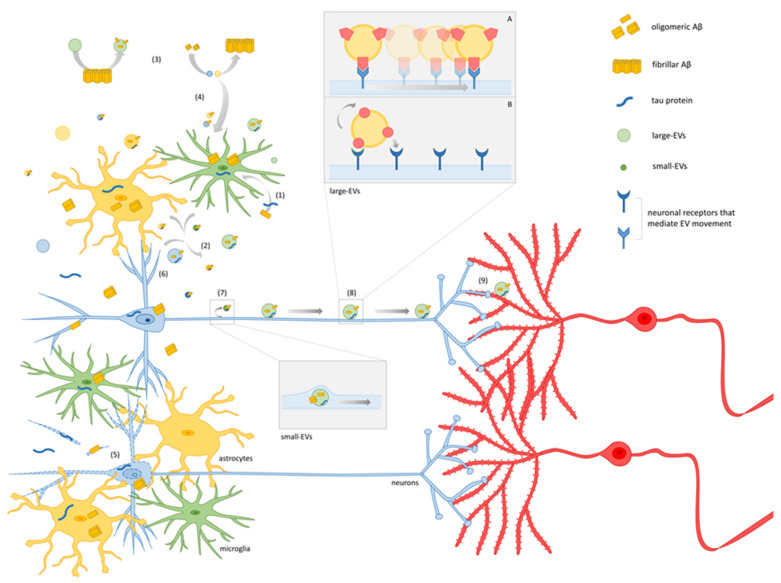
Mechanisms of EV-mediated propagation of synaptic dysfunction. Scheme of the onset and propagation of synaptic dysfunction in vulnerable brain regions in the early phases of AD. The model is described in the text section “A model for EV-mediated propagation of synaptic dysfunction”. Parts of the figures were drawn by using pictures from Servier Medical Art, licensed under a Creative Commons Attribution 3.0 Unported License (https://creativecommons.org/licenses/by/3.0/; access date 20 November 2022). Part of the figure is modified from [99], distributed under the terms of the Creative Commons CC-BY license (https://creativecommons.org/licenses/by/4.0/; access date 20 November 2022).

Microglia large EVs have been shown to promote the solubilisation of Aβ aggregates, thus shifting equilibrium from an almost inert insoluble form of the peptide toward soluble and neurotoxic species [21,22,92] (Figure 2). In addition, when exposed to Aβ 1–42, microglia release large EVs already carrying neurotoxic Aβ species on their surface and in their lumen [17,21,22]. Once injected into the mouse EC, these Aβ-loaded EVs (Aβ–EVs) reduce synaptic transmission and consequently inhibit LTP. Interestingly, these effects are first detected in the vicinity of the injection site, but synaptic dysfunction then propagates from the EC to the hippocampus [17]. The spreading of synaptic dysfunction was ascribed to the ability of Aβ–EVs to move on the neuron surface, along axonal projections connecting the EC to the DG (Figure 2). Indeed, when Aβ–EV motility was inhibited, no propagation of LTP deficit along the entorhinal–hippocampal circuit occurred [17]. Although it has been reported that tau can be released inside microglia large EVs [93], no data are currently available on the role of such EVs in tau spreading.

Supporting an interplay between tau and Aβ in AD pathogenesis, the release of small tau-carrying EVs is higher from microglia surrounding Aβ plaques than phagocyte hyper-phosphorylated plaque-associated tau, as well as from apoptotic neurons and synapses [55]. Notably, microglia phagocyte AD misfolded proteins and apoptotic structures aiming at their clearance [94], and exploited EV release at least in part as a disposal mechanism [17,21], as other cells do [25,95,96,97]. 

In accordance with all these studies, large EV production from myeloid cells (microglia/macrophages) is very high in AD patients and correlates with white matter lesions and hippocampal atrophy in prodromal AD, the preeminent expression of neuronal damage in the human brain [98]. In addition, the neurodegenerative microglia signature is enriched in brain-derived small EVs from CAST.APP/PS1 AD mice [30].

### 3.2. EVs Released by Astrocytes

Astrocytes play important roles in neuronal support, maintaining brain homeostasis of ions and neurotransmitters. They represent a fundamental component of the synapse, being part of the so-called “tripartite synapse” together with the pre-synaptic terminal and the post-synaptic compartment [100,101]. Astrocytes are involved in synapse formation, can regulate synaptic transmission, and can also eliminate synapses. Accordingly, similarly to microglial EVs, large EVs released by astrocytes promote excitatory synaptic transmission [86] and move extracellularly, inducing spine formation at sites of stable contact [102], while small EVs carry the neuroprotectant neuroglobin [103], promote neurite outgrowth and neuron survival, and also stimulate synaptic transmission and formation [104,105]. Nevertheless, upon interleukin 1β exposure of donor rat or human primary astrocytes, released small EVs undergo neuronal uptake more frequently than EVs from control cells and are able to inhibit neurite outgrowth, neuronal branching, and firing [104,106].

Like microglia, astrocytes are central players in AD pathology [3] and show early changes in the disease [77,107]. Those close to dystrophic neurites or Aβ plaques alter their morphology, becoming hypertrophic or atrophic [108], as well as their gene and protein expression, displaying a heterogeneous range of activation states [109,110]. In tauopathies, mouse model astrocytes display early functional deficits and lose their neuro-supportive function [111]. In addition, tau accumulation in astrocytes of the DG of the hippocampus, a phenomenon also found in the brain of AD-affected individuals, has been found to cause neuronal dysfunction and memory deficits in mice [112].

Astrocytes are very efficient in engulfing dead cells, synapses, and protein aggregates (e.g., of Aβ) [113,114,115,116,117,118,119], and astrocytes with high Aβ load are frequently found in the AD-affected brain [120]. However, as opposed to microglia, astrocytes are extremely inefficient at degrading phagocytosed material [121], including Aβ 1–42 protofibrils [122]. Aβ accumulation in astrocytes over avery long time further affects endosomal and lysosomal function and induces the release of EVs carrying Aβ (in its N-terminal truncated form) and ApoE to favour elimination of undegraded materials [66,122,123,124]. Furthermore, the Aβ 1–42 proxy Aβ 25–35 induces phosphorilated-tau overproduction in human astrocytes in culture and increases its release within small EVs [24]. EVs carrying Aβ/phosphorylated tau are neurotoxic, causing synaptic loss, axonal swelling, vacuolization of neuronal cell bodies, severe mitochondrial impairment, cholesterol deposits in lysosomal compartments, and apoptosis [66]. 

The first evidence of the involvement of EVs released by astrocytes in AD progression came from the finding that, in response to Aβ, astrocytes release small EVs containing prostate apoptosis response 4 (PAR4) and ceramide, which induce apoptosis in other astrocytes upon internalization, likely contributing to neurodegeneration [67]. Interestingly, vesicular ceramide was later found to be responsible for astrocyte small EVs’ ability to aggregate Aβ peptides [125] (Figure 2). Subsequently, phosphorylated tau and proteins of the Aβ 1–42 peptide-generating system were found in astrocyte-derived small EVs extracted from the plasma of AD patients [28] as well as various complement proteins that are central players in synaptic pruning [27,126]. In line with this evidence, when isolated from AD patients, astrocyte EVs were more efficient than neuron EVs in inducing complement-mediated neurotoxicity and in reducing neurite density and decreasing cell viability in either cultured neurons or human iPSC-derived neuron-like cells [68].

Additional proofs of the implication of astrocyte EVs in AD progression came from: (i) the enrichment in astrocyte-derived molecules in AD EVs compared to EVs from mild cognitive impairment (MCI) patients [18,127], and (ii) the most significant association of a protein module enriched in astrocyte-specific EV markers with AD pathology and cognitive impairment compared to the proteome of other brain cell-derived AD EVs [128].

Despite extensive evidence suggesting important roles for EVs released by astrocytes in AD synaptopathy evolution, further studies will be necessary to gain a clearer understanding of their early action on the synapse.

### 3.3. EVs Released by Neurons

During development, neural stem cells can secrete EVs capable of affecting the proliferation and differentiation of neighbouring cells through the propagation of specific miRNAs able to reprogram multiple cellular mechanisms in recipient cells [129,130]. In the mature nervous system, neurons maintain their ability to produce EVs and use these vesicles to communicate with other cells and to regulate several phenomena such as homeostasis, immune response, and synaptic plasticity [131,132]. Although neuronal-derived EVs have been shown to interact with glial cells and affect microglia phagocytic activity [133] and the expression of the glutamate transporter GLT1 in astrocytes [134], in vitro studies suggested that EVs secreted by cortical neurons preferentially bind to other neurons [135], allowing neuron-to-neuron diffusion of specific cargoes. In addition, EV release has been shown to be strongly modulated by synaptic activity [136,137,138].

Neuron-derived EVs could be differentiated from the ones produced by other cell types by the expression of specific markers, such as the L1 cell adhesion molecule (L1CAM), the GluR2/3 subunits of the glutamate receptors, and the GPI-anchored prion protein [136,139]. However, the prion protein was also later identified in astrocyte-derived EVs [102,140].

Given their ability to move from cell to cell, neuronal EVs have been hypothesized to be able to spread along a neural network of connections in a trans-synaptic manner and contribute to the propagation of misfolded proteins in neurodegenerative diseases such as AD. 

Indeed, the amyloid precursor protein (APP) and its metabolites, including the Aβ peptide, have been shown to be secreted within neuron-derived small EVs [141,142,143,144,145]. In addition, Sardar Sinha and colleagues [26] demonstrated that the impairment of the formation/secretion of small EVs can suppress the diffusion of Aβ oligomers to other neurons. 

Interestingly, as opposed to microglial large EVs, neuronal and neuroblastoma cell line small EVs seem to promote amyloidogenesis of soluble Aβ through the binding of the amyloid peptide to the glycosphingolipid glycans [61,62,92] and to the cellular prion protein (PrP^c^) [146,147] present on their surface. Neuronal small EVs contain higher levels of glycosphingolipid glycans in their membrane compared to small EVs secreted by other cell types, and this significantly increases Aβ affinity for neuronal-derived EVs [63,148]. The interaction between the Aβ peptide and neuronal EVs can lead to accelerated Aβ fibril formation and, therefore, drive conformational changes in the Aβ to form nontoxic amyloid fibrils [61]. Indeed, small EV markers such as Alix have been observed to be concentrated in senile Aβ plaque in AD brains [141]. Furthermore, PrP^C^ on small EVs negatively regulates Aβ 1–42 uptake by neuronal cells [147] while, on the other hand, neuron-derived small EVs can be efficiently internalized by microglia and promote Aβ degradation, suggesting an overall protective effect of neuron-derived EVs against AD pathology [61,149], as opposed to microglia large EVs [21]. This suggests that neuronal small EVs and microglia large EVs may play very distinct roles in neurodegeneration. In agreement with this hypothesis, protective effects against Aβ-induced pathology and synaptic transmission have been observed following chronic administration of small EVs derived from neuroblastoma cells or primary neurons in the hippocampus [62,63], and a significant rescue of Aβ-induced LTP impairment has been observed after the intracerebroventricular infusion of small EVs in rats [60], strengthening the link between neuronal-derived EVs and neuroprotection. In contrast, EVs released from cultured human neurons and cell lines harbouring familial AD presenilin 1 mutations show neurotoxicity towards cultured wild type neurons in terms of intracellular calcium regulation, mitochondrial functions, and sensibility to excitotoxicity [25].

By means of immunoassays specifically designed to detect the full-length tau protein, considered to be the aggregation-competent form, Guix and colleagues [150] revealed that small EVs secreted by human iPSC-derived neurons or present in human biofluids are highly enriched in full-length tau compared to the extracellular solution, indicating that neuronal EVs carry aggregation-competent tau proteins. In addition, neuronal small EVs could mediate the trans-synaptic propagation of tau protein regardless of its phosphorylation state, in an activity-dependent manner [59], but unfortunately their neurophysiological correlates in vivo have not been explored yet. Interestingly, in analysing neuronal EVs isolated from the plasma of AD and frontotemporal dementia patients, a correlation between the vesicular levels of some synaptic proteins and patients’ cognitive status have been defined, mirroring the decrease in synaptic proteins and the synaptic dysfunction in the affected brain [151,152].

### 3.4. Mixed EV Populations Isolated from Body Fluids or Brain Tissue

In an increasing number of papers, small EVs isolated from the interstitial space of brain tissue or body fluids (mainly CSF and plasma) have been investigated. These samples represent a real liquid biopsy of the system (animal or human) they are coming from and a window on the microenvironment of specific tissues/compartments in these organisms in a particular situation (e.g., stage of pathology). For this reason, these specimens have been particularly useful for the study of biomarkers for the diagnosis and prognosis of different diseases, including AD and related cognitive defects [27,28,29,31,95,153,154,155,156]. On the other hand, EVs from brain tissue and body fluids are mixed populations of EVs of different cell origins, and the extraction of cell-type-specific EVs is possible only after an additional step of immunoisolation (e.g., in [32]). 

As mentioned above (EVs released from astrocytes), small EVs isolated from AD brains typically express more glia- than neuron-derived molecules compared to EVs from healthy subjects [29], and those isolated from the brain of AD mouse models indicate that Aβ can be processed and oligomerized in EVs [157]. Studies on small EVs from human plasma and CSF corroborated the prevalent exposure of Aβ peptides on the surface of EVs [25,158]. A fascinating hypothesis is that binding to the EV surface may be the basis for the low CSF levels of Aβ 1–42 that typically correlate with AD [92]. On the other hand, small oligomeric globular tau, together with other isoforms, phosphorylated or not, have been found inside EVs from tauopathy mice models and AD patients [15,18,23,26,29,31,59] and display an elevated tau seeding activity [18,59,65]. Tau particles have been visualized in the inner leaflet of the EV membranes by electron microscopy [15], and the exposure of even a small portion of tau oligomers on the outer membrane leaflet is highly controversial [18]. 

Joshi and colleagues were the first to report that EVs isolated from the CSF of AD patients affect neuronal calcium homeostasis and are neurotoxic [21]. A subsequent study showed that small EVs from AD CSF samples are internalized by neurons and affect mitochondrial function, making the cells more vulnerable to excitotoxicity [25]. After internalization, small EVs can be degraded into lysosomes or transfer their content to the cytosol. However, two independent studies recently revealed that small EVs from mouse models and AD patients can avoid disassembly and, still intact, can transport Aβ and tau in an anterograde manner along axons and migrate trans-synaptically to a connected neuron in vitro [26,64] (Figure 2). Interestingly, this might occur also in vivo: small EVs isolated from the plasma of healthy mice and injected into the DG of the hAPP-J20 AD mouse model were engulfed by microglia surrounding Aβ plaques. However, a fraction of them not engulfed by microglia propagated through the hippocampus and up to the cortex in 20 days [159]. Nevertheless, the use of lipophilic dyes to visualize EVs in vivo represents a significant weakness of this study, as explained in the next section.

A first clue that small EVs are able not only to spread throughout the brain, but also to induce and propagate neurophysiological dysfunction in the AD brain came from a study from Dr. Ikezu’s laboratory. In this work, Ruan et al. showed significant spreading of abnormally phosphorylated tau in both the contralateral and ipsilateral hippocampus 4.5 months after inoculation of EVs derived from the brain of prodromal AD and AD patients in the outer membrane layer of the DG [18]. Unexpectedly, tau was mainly found in the GAD67+ interneurons and GluR2/3+ mossy cells in the hilus region of the hippocampus. On the other hand, tau oligomers and fibrils isolated from the same subjects and injected in equal amounts caused very limited tau pathology. Importantly, these phenomena were associated with intrinsic synaptic dysfunction of CA1 pyramidal neurons and reduced input from interneurons and were mediated by tau seeding caused by inoculated EVs.

## 4. A Model for EV-Mediated Propagation of Synaptic Dysfunction

The study of EV-neuron interaction is fundamental in order to clarify the molecular mechanisms underlying the contribution of EVs to neurodegeneration and to find potential therapeutic targets. Studying the interaction of EVs with target cells in vivo is still difficult due to technical limitations (e.g., low visibility of fluorescent EVs in complex mouse tissue and restricted extracellular space in the brain) and high aspecificity risk associated with EV labelling (i.e., lipophilic dyes such as PKH67, DiR/DiD, MemGlow, and mCling can create EV-like micelles or incidentally label non-EV particles) [160]. 

The research pioneered by Stefan Momma’s laboratory represents an exception, as his team was able to show the transfer of functional mRNA (Cre mRNA) from hematopoietic EVs to neurons in vivo, taking advantage of a Cre-LoxP recombinase system [161]. Furthermore, the expression of fluorescent EV tags, e.g., CD9-GFP or mEmerald/CD9, under cell-specific promoters in transgenic mice or after lentiviral vector injection is rapidly catching on for in vivo imaging of EVs [55,162], which might include future monitoring of Aβ/tau-carrying EVs in the brain.

However, so far, most of the knowledge on glial EV–neuron interaction comes from in vitro imaging studies, some of which exploit microfluidic or optical manipulation approaches. 

Combining such imaging studies with in vivo electrophysiological analyses, we could outline a model for EV–neuron interaction and the spreading of pathological EV cargoes in AD (Figure 2). Following this model, Aβ and tau start to accumulate intracellularly in vulnerable brain regions (1) and are then released by neurons and glia in association with EVs, generating Aβ/tau-carrying EVs (2). In addition, extracellular Aβ and tau bind to lipids at the surface of EVs already present in the pericellular space [61,62,63,92] (3), increasing the pool of Aβ/tau-carrying EVs. Microglial cells can take up Aβ upon endocytosis of neuronal small EVs storing Aβ [61,62] (4) or upon phagocytosis of cytopathic cells or cell debris [55,94] (5), at a later stage. 

EVs loaded with Aβ/tau first affect the synapse locally (at the site of EV release or interaction with extracellular Aβ/tau (6)). Then, small EVs are internalized by neurons and travel inside axons, exploiting intracellular trafficking mechanisms [64,163] to trans-synaptically spread their Aβ/tau cargo (as described by [26,59,64] (7)). On the the other hand, larger EVs, too big to be taken up and travel inside axons, move on the neuron surface along axonal projections to reach connected neurons [17,99,102] (8)). Once they reach synaptic sites on dendrites, large EVs can signal locally and/or can be internalized to deliver their cargoes (9). A jump may be necessary for large EVs to transit from the surface of one neuron to a connected one, a phenomenon that occurred in our in vitro studies [102].

This bi-modal mechanism of interaction between small and large EVs and neurons is supported by optical manipulation experiments combined with in vitro fluorescence analysis of EV–neuron contact. Large EVs placed on cultured neurons by optical tweezers and moving on the neuron surface are not internalized by neurons [164] and can be recaptured by the laser trap, which stops their extracellular motion [102]. However, these EVs can undergo partial fusion with the neuron plasma membrane, as indicated by live monitoring of fluorescence intensity of EVs labelled by a dequenching dye, making possible the transfer of EV cargo, such as miRNAs, into neurons [90].

Post-fixation analysis of neurons exposed to fluorescent EVs in bulk provided further information on the fate of small and large glial EVs on neurons: most large EVs (mCLING-labelled) are taken up inside cell somata (94%, [102]) and large dendrites (82%, [102]), but not inside axons (i.e., thin ≤2 μm in diameter neurites in cytoplasmic-RFP-transfected neuronal cultures; 6% astrocyte large EVs, [102]; 3% microglial large EVs, [17]). On the other hand, the internalization of small EVs (lipophilic dye-labelled) of various cell origins into neurons has been largely proven (PKH, [26,33,165]; mCling [140]; DiO, [58]), as well as their transport inside axons for trans-synaptic transfer of their cargo [26,59,64].

### Molecular Mechanisms Underlying EV–Neuron Interaction

Insights into the molecular mechanisms underlying EV–neuron interaction come from a few studies. Brenna et al. reported that the cellular prion protein (PrP^c^), a GPI anchor protein highly enriched in brain EVs [139,140,166], may modulate small EV internalization. Specifically, the presence of PrP^c^ on the surface of astrocyte-derived small EVs limits EV uptake by primary neurons [140]. In agreement with that, we reported that PrP^c^ is enriched on the surface of astrocytic EVs and showed that PrP^c^ interaction with its receptor(s) on neurons favours motion of large EVs at the neuronal surface [102]. In more detail, we found that vesicular PrP^c^ mediates the binding of large EVs to a neuronal receptor (PrP^c^ interacting molecule) that is coupled to a dynamic actin cytoskeleton, thus eliciting passive transport of EVs on neurons [102] (Figure 2 insert A). Only a small fraction of large EVs seem to be able to actively move on the neuron surface, taking advantage of the presence inside their lumen of actin filaments and ATP as their energy source [102,167] (Figure 2 inset B). 

A recent study also supported the implication of surface transglutaminase 2 (TG2) in the interaction between astrocytes-derived EVs and neurons [168]. TG2 modulates neuronal calcium homeostasis, synaptic transmission, and cell adhesion. In addition, it interacts with Aβ and other proteins involved in AD, is implicated in Aβ processing, and is over-expressed in AD [169]. Among TG2 interactors involved in cell adhesion, transmembrane proteoglycans, also called syndecans, were shown to mediate adhesion of microglial EVs to neurons (through their heparin sulphate chain [90]). Thus, vesicular TG2 may represent the interactor of neuronal syndecans and contribute to a stable EV–neuron contact.

That glycans have a role in EV cellular uptake is known [170,171]. Galectin is another molecule connected to EV–cell interaction [172], together with integrins, which can bind a plethora of molecules, including intercellular adhesion molecules (ICAMs, [173]) or extracellular matrix (ECM) components on the surface of target cells [174,175,176]. In fact, blocking integrin-mediated interactions partially suppresses astrocyte EV uptake by neurons [106]. 

Finally, phosphatidylserine (PS), a phospholipid typically exposed on the outer leaflet of the EV membrane [177], also participates in the interaction between glial EVs and neurons. Cloaking PS residues with annexin-V decreases adhesion of astrocytic large EVs to neurons [90] while promoting that of microglial large EVs [17]. Additionally, the treatment largely limits the motion of microglial large EVs in vitro at the neuronal surface and blocks EV-mediated spreading of synaptic dysfunction in vivo, without affecting localized EV action [17]. 

Proteomic studies in EVs isolated from mouse AD brain cells [30,58,178], human AD specimens [29,32,95,127], and iPSC-derived neural cells [128] confirmed changes in the expression of adhesion molecules (PrP^C^, integrin-β1, -β2, -αx, annexin A5, A6, A7, neuroligin, Thy-1, CCL2, APP, and ApoE), and also showed alterations in molecules involved in actin cytoskeleton dynamics (glicoprotein M6-A, formin-like protein-1, the puromycin sensitive aminopeptidase interacting with ezrin, and an ERM protein crosslinking actin filaments with plasma membranes), or those interacting with microtubules (e.g., α-synuclein [179]). Canonical pathways of small EV proteins from human AD CSF include various endocytic pathways, virus entry, cell adhesion, and actin cytoskeleton signalling [127]. Interestingly, APP and PrP^c^, which are able to interact with each other and with many other proteins, including Aβ, tau, the ECM, adhesion molecules, cell cytoskeleton [41,180,181,182,183,184,185], are significantly co-upregulated in brain EVs from AD patients compared to age-matched controls specifically in the preclinical stages of the disease, suggesting their implication in disease onset and/or early development [95]. Notably, APP has been reported as necessary for Aβ and tau oligomers to enter the neurons and induce abnormal synaptic function and memory [41,186]. 

Therefore, all the adhesion molecules interacting with the cell cytoskeleton that are differentially expressed in AD EVs may influence EV–neuron contact and signalling. In accordance, small EVs isolated from the frontal cortex of AD patients display an increased uptake by neurons (coupled with enhanced tau transfer efficacy) compared to EVs from prodromal AD and control subjects [18]. In contrast, microglial large EVs carrying Aβ are more prone to extracellular motion on axons, and they move faster and preferentially towards the cell periphery compared to EVs not carrying Aβ [17]. However, it remains largely unknown which surface proteins are implicated in the alteration of EV–neuron interactions. 

It should be also taken into consideration that, to move extracellularly in the brain parenchyma, EVs have to interact with the ECM. Not surprisingly, several EV surface molecules act on or interact with the ECM [187,188,189,190] (e.g., metalloprotease and others), while ECM components can associate with the EV membrane [191]. The ECM is involved in several brain processes, including synaptic plasticity [192], and is altered in AD, especially regarding the perineuronal nets that form net-like structures around neurons [35], thus potentially affecting EV extracellular motion.

## 5. Discussion

Loss of synaptic functionality is the earliest event in AD pathology. However, little is known about how synaptic dysfunction starts and propagates along neuronal circuits to cause network abnormalities. The role of EVs of different origins as vehicles for Aβ and tau spreading throughout the brain and across the synapse is widely accepted. So far, only a few papers have examined the ability of EVs to effectively induce and propagate AD-related neurophysiological alterations in the brain [15,17,18]. These studies highlight a major role for both small and large EVs in these processes, placing them at the top of the list of putative targets for novel therapies. 

While we still lack a direct proof of EV motion in vivo, in vitro studies provided us with some clues to build a model for EV-mediated propagation of Aβ/tau and related synaptic deficits (Figure 2). In this model, large and small EVs interact with neurons in distinct ways, spreading dysfunction by moving at the axonal surface or inside neuronal processes, respectively.

The challenge is now to decipher the molecular mechanisms underlying EV–neuron interaction and EV motion inside/outside neuronal processes. This will represent a significant step towards the establishment of novel therapeutic strategies to hamper AD progression. Notably, these mechanisms may be shared by other neurodegenerative diseases characterized by the spreading of misfolded proteins along neuronal connections, such as Parkinson’s disease and amyotrophic lateral sclerosis (ALS).

It is worth noting that, while the current knowledge about EV action on the synapse in AD comes from the analysis of EVs of either multiple cell origins or derived from microglia and only marginally astrocytes and neurons, still unexplored is the role of EVs released by oligodendrocytes, which typically exert neuroprotective functions aimed at preserving brain homeostasis [193], and the role of other brain cells, such as vascular and blood–brain–barrier (BBB) epithelium cells, which may play some roles in the evolution of the pathology. Furthermore, the large majority of the studies regarding AD focus on small EVs, although recent evidence reveals that large EVs deserve more attention as feasible pharmacological targets. In addition, Aβ-tau interplay, which notably magnifies neuronal circuit impairment [47], is something that would be worth looking into more deeply in relation to AD EV action.

Relative to the mechanisms of EV-mediated induction of synaptic failure, it should be pointed out that EVs may cross the BBB [161], and systemic inflammation may also modulate synaptic function via blood EVs. This is somewhat suggested by the evidence that intracerebroventricular injection of intestinal small EVs from intestinal ischemia/reperfusion mice causes microglial activation, neuronal loss, synaptic stability decline, and cognitive impairment [194]. Moreover, the exposure of neurons to EVs derived from systemic autoimmune-driven diseases such as rheumatoid arthritis leads to synaptic dysfunction, as evidenced by electrophysiological recordings of mEPSCs and analysis of synaptic markers [195]. Investigating the mechanisms regulating EV entrance in the brain will be instrumental to clarify the role of peripheral EVs in AD synaptic failure.

Finally, small EVs of different origin have been proven as potential therapeutic agents in AD models. Aside from the beneficial action of neural and mesenchymal stem cell-derived small EVs, which mirror the well-known immunomodulatory and therapeutic power of their donor cells [196], neuronal EVs showed protective and curative effects against Aβ-induced pathology and synaptic alterations. In addition, understanding how EVs of oligodendrocyte and vascular cell origin influence synaptic transmission in AD may provide new options for EV-mediated therapeutic applications in the disease.

## 6. Conclusions

To conclude, recent findings point to a substantial role for large and small EVs of different cell sources in the onset and propagation of synaptic alterations in early AD. Given the lack of effective therapies for this terrible disease, decoding the molecular mechanisms at the basis of the biological action of EV will be crucial in developing novel strategies to limit the progression of neurodegeneration.
